# Cross communication of diet-microbiome-immune interactions in cancer immunotherapy

**DOI:** 10.1016/j.xcrm.2022.100806

**Published:** 2022-11-15

**Authors:** Cheng-Bei Zhou, Jing-Yuan Fang

**Affiliations:** 1 Division of Gastroenterology and Hepatology, Renji Hospital, School of Medicine, Shanghai Jiao Tong University, Shanghai, China; 2 Shanghai Institute of Digestive Disease, Shanghai, China; 3 State Key Laboratory for Oncogenes and Related Genes, Key Laboratory of Gastroenterology & Hepatology, Ministry of Health, 145 Middle Shandong Road, Shanghai 200001, China

## Abstract

Simpson et al. have highlighted a diet-driven microbiome community, which paves the way for landmark therapeutic avenues for facilitating efficacy and minimizing immune-related adverse events during neoadjuvant immune checkpoint inhibitor treatment in melanoma patients. This innovative attempt inspires application of the diet-microbiome-immune interaction axis to maximize clinical benefits.

## Teaser

Simpson et al. have highlighted a diet-driven microbiome community, which paves the way for landmark therapeutic avenues for facilitating efficacy and minimizing minimizing immune-related adverse events during neoadjuvant immune checkpoint inhibitor treatment in melanoma patients. This innovative attempt inspires application of the diet-microbiome-immune interaction axis to maximize clinical benefits.

## Main text

In the latest issue of *Nature Medicine*, Simpson et al. demonstrated several findings of great significance and potential clinical implications.^1^ Promisingly, they took the lead to explore the beneficial effect of gut microorganisms associated with high intake of fiber and omega-3 fatty acids in improving neoadjuvant immune checkpoint inhibitor (ICI) outcomes in high-risk resectable metastatic melanoma patients, involving promoting anti-tumor immune responses and minimizing immune-related adverse events (irAEs). The higher immune response rate and less severe toxicities wwere correlated with *Ruminococcaceae*, *Akkermansia muciniphilia*, and methanogenic-archaea-dominated microbiome, which was partly linked with higher fiber and omega-3 consumption. Importantly, they presented a framework to prove that the distinctions in microbial signatures of immunotherapy response and irAEs could be explained by the underlying variance in intestinal microbial community ecology, which would be affected by various environmental inputs, e.g. diet, independently of geographical location. This responds to the theory that individual microbiome polymorphism has been listed as one of the ten major characteristics of cancer.^2^ The major innovations of this study have made these findings more convincing and expanded prospective therapeutic approaches. From the perspective of study design, they enrolled large, prospective, multi-regional, highly homogeneous cohorts in which patients received a combination of PD-1 and CTLA-4 blockade in a prospective clinical trial. They also introduced maneuverable dietary factors between microbiota and efficacy/irAEs during immunotherapy. With the support of integration of sequencing analysis, microbial functional analysis, and machine learning, they originally outlined microbiome community and further set up a path to develop more targeted interventions in remodeling gut microbiome for treatment outcome improvement.

With the advent of cancer immunotherapy in recent years, our team and other scientists keep exploring interventions to reduce severe irAEs. The main influencing factors of heterogeneous clinical response to immunotherapy include gut microbiome, diet, immune microenvironment, genetic susceptibility factors, environmental exposure factors, etc. Both the microbiota and the diet impact immunotherapy outcomes independently. The robust commensal microbiota can facilitate cultivation of immune tone before and during immunotherapy. Multiple research efforts have highlighted that microbial community could regulate patients’ sensitivity to immunotherapy through interacting with both endogenous and exogenous metabolites, leading to crosstalk between metabolic network and immune microenvironment, thereby impacting anti-tumor immune response and toxicities.^3^ Currently, it has been confirmed in many clinical cohorts and preclinical models that fecal microbiota profiles can affect immunotherapy efficacy and toxicities in various solid tumors through various approaches.^4^

Diet is a noteworthy factor that contributes to shape microbial structure and function, individual differences in which propel distinct impacts of certain nutrients on host metabolism. Accumulating evidence from animal models, epidemiology, and clinical studies has shown that dietary factors, as one of the major gut microbiome determinants, play a critical role in cancer prevention, prognosis, and treatment including chemotherapy, radiotherapy, and immunotherapy.^5^ Sufficient dietary fiber is shown to remarkably improve the progression-free survival (PFS) of patients receiving ICIs.^6^ Currently, there are clinical and preclinical studies looking at the impact of supplements, e.g. diet, on the outcomes of ICIs. However, to some extent, individual responses to specific dietary factors depend on the baseline microbial community, implying intricate diet-microbiota-metabolome-immunity crosstalk.^7^ Dietary nutrients, e.g. fiber, vitamin D, and omega-3 fatty acids, affect patients’ response to immunotherapy partially via metabolome-microbiome-immunomodulation. Fiber has been shown to be immunomodulatory, with growing evidence that short-chain fatty acids (SCFAs) modulate T cell differentiation, modulate T effector/regulatory T cell balance, and enhance the memory potential of activated CD8 T cells.^6^ In a preclinical study, the consumption of a high-fiber diet to modulate baseline gut microbiome can trigger STING-type I IFN-dependent monocyte reprogramming in tumor microenvironment, thus improving the clinical outcome of ICIs.^8^

So far, scientists have found that diet-commensal microbiota intervention strategy has achieved some success in chemotherapy and radiotherapy. Tumor patients with a high-fiber habitual diet seem to develop enriched pro-response and fiber fermenting microorganisms, and thus are more likely to respond to immunotherapy. Data from the US cohort no.2 in this study was the result of a pioneer in the field of dietary/probiotic intervention during immunotherapy.^6^ Preclinical models showed impaired response to ICIs in mice fed with lower-fiber diet or probiotics with IFN-g T cell response suppression.^6^ In the present study, patients who lack fiber-imprinted microbiomes and enhance their dietary fiber receive more clinical benefit from the immunotherapy setting. Therefore, further investigation on overcoming the variety of dietary patterns and targeting the variation of microbial communities is crucial to design personalized interventions to optimize immunotherapy outcomes. The diet-microbiota-immunity axis has potentially been regarded as a vital intervention and an interesting perspective on clinical application. In the approaching future, individualized methods for identifying the ideal “bacterial” responder signature may be required, including the utilization of diet, probiotics, antibiotics, fecal microbiota transplantation (FMT), bacteriophages, and other precise interventions, as well as the incorporation of internal/external factors of cancer, to modulate the microbiome and induce a microbiome and immune landscape favorable to treatment responses.^9^^,^^10^ Among them, diet intervention seems to be a preferred solution due to its safe profile and low cost, whereas randomized controlled trials are lacking up to present. The most prominent challenge we are facing is how we can recognize responders and take the best advantage of the intricate and profound diet-microbiota-immunity axis to safely, effectively, and fully unleash the therapeutic potential and minimize irAEs during immunotherapy. This needs to be implemented in a larger, diverse cohort consisting of patients with earlier stages of cancer. In general, the successful attempt in this study has provided us insights into the strategy of microbial combination pool development based on adjunctive strategies as precise dietary regimens to broaden beneficial bacteria or starve harmful bacteria, demonstrating a path to optimal immunotherapy outcomes.
